# Kardioonkologie: Herzdosis während einer Radiotherapie im Thoraxbereich

**DOI:** 10.1007/s00066-020-01733-6

**Published:** 2021-01-05

**Authors:** Marciana-Nona Duma, Andrea Wittig

**Affiliations:** Klinik für Strahlentherapie und Radioonkologie, Universitätsklinikum Jena, Bachstr. 18, DE-07745 Jena, Deutschland

## Hintergrund

Die Dosis am Herzen ist bei der thorakalen Radiotherapie (TRT) des nichtkleinzelligen Bronchialkarzinoms (NSCLC) mit einer Übersterblichkeit korreliert. Es ist jedoch oft nicht möglich, das ganze Herz zu schonen. Daher war das Ziel der hier zu kommentierenden Studie, kardiale Substruktur(en) und Dosisschwellenwerte zu definieren, deren Unterschreitung die frühe Letalitätsrate wegen Herztoxizität senken kann.

## Patientengut und Methode

14 kardiale Substrukturen wurden an 5 Template-Patienten mit repräsentativer Anatomie konturiert. 1161 NSCLC-Patientendaten wurden nicht rigide auf diesen 5 Template-Patienten registriert und ihre Strahlentherapiedosis aufgezeichnet. Die mittlere und maximale Dosis für jede Substruktur wurden extrahiert und die Mittelwerte für Vorhersagemodelle (Elastic-net-LASSO- und Random-40-Forest-Survival-Modell) bestimmt. Beide Modelle wurden optimiert, um Variablen zu extrahieren, die am meisten zum Gesamtüberleben beitragen. Und die Modellkoeffizienten wurden ausgewertet, um diese Substrukturen auszuwählen. Die wichtigsten Variablen beider Modelle wurden ausgewählt und in multivariablen Cox-proportionalen Hazard-Modellen ausgewertet. Es wurden eine Schwellendosis definiert und Kaplan-Meier-Überlebenskurven erstellt.

## Ergebnisse

978 Patienten verblieben nach der visuellen QA in der Registrierung. Die Rangfolge der Modellkoeffizienten wählte die maximale Dosis für den rechten Vorhof, die rechte Koronararterie und die Aorta ascendens als die wichtigsten Regionen, die mit dem Überleben assoziiert sind. Die maximale Dosis für die ausgewählte Herzregion zeigte Signifikanz im multivariablen Model (Hazard Ratio 1,01 Gy, *p* = 0,03) nach Berücksichtigung des Tumorvolumens (*p* < 0,001), des N‑Stadiums (*p* < 0,01) und des Performance-Status (*p* = 0,01). Der Schwellenwert für die maximale Dosis (als Äquivalenzdosis in 2 Gy-Fraktionen – EQD2) lag bei 23 Gy. Kaplan-Meier-Überlebenskurven zeigten einen signifikanten Split (Log-Rang *p* = 0,008).

## Schlussfolgerung der Autoren

Die maximale Dosis für die Herzregionen rechter Vorhof, rechte Koronararterie und Aorta ascendens hat den größten Einfluss auf das Überleben der Patienten. Es wurde eine maximale EQD2 von 23 Gy ermittelt, die in zukünftigen Studien als Dosisbegrenzung in Betracht gezogen werden sollte.

## Kommentar

Die meisten Todesfälle weltweit sind auf kardiopulmonale und onkologische Erkrankungen zurückzuführen [1]. Eine onkologische Therapie kann aber zusätzlich eine kardiopulmonale Belastung sein und negative Auswirkungen auf die Lebenserwartung haben. Die aktuelle, hier kommentierte Studie versucht anhand eines großen Kollektivs mit einer innovativen Technik („image-based data mining“ und Random-forest-survival-Modell) die Frage nach der optimalen Konturierung des Herzens bei der TRT zu beantworten. Die Autoren konnten in ihrer Analyse zeigen, dass der rechte Vorhof, die rechte Koronararterie und die Aorta ascendens prognostisch am wichtigsten sind. Sie empfehlen, eine D_max_ EQD2 von 23 Gy in der Planung einzuhalten.

Der größte Teil unserer Kenntnisse über den Zeitpunkt und die klinischen Manifestationen der therapieassoziierten „radiation-induced heart disease“ (RIHD) stammt aus Daten nach Strahlentherapie „gutartiger“ Erkrankungen sowie des M. Hodgkin und des Mammakarzinoms. Im Allgemeinen wurden diese Patienten in einem jüngeren Alter behandelt und lebten lange genug, um die kardialen Veränderungen Jahrzehnte nach der Radiotherapie (RT) aufdecken zu können. Deshalb wurde die symptomatische RIHD als eine Langzeitnebenwirkung betrachtet, die erst Jahrzehnte nach der RT auftritt und somit kein signifikantes Risiko für Patienten mit lokal fortgeschrittenen Neoplasien und schlechten 5‑Jahres-Überlebensraten darstellt.

Jedoch wurde in den letzten Jahren bei Patienten mit NSCLC, die eine Strahlentherapie erhielten, die Herzdosis mit einer höheren Letalität korreliert. Patienten, die im Rahmen der RTOG-0617-Studie dosiseskaliert mit einer Gesamtdosis von 74 Gy behandelt wurden, hatten nur eine mittlere Überlebenserwartung von 20,3 Monaten im Vergleich zu 28,7 Monaten nach einer Gesamtdosis von 60 Gy im Standardarm. In der Analyse wurde die Herzdosis als ein Risikofaktor für die erhöhte Sterblichkeit identifiziert [2]. Das Ergebnis der Studie weist somit ebenfalls eine relevante kardiale Morbidität und sogar Letalität bereits 2–3 Jahre nach TRT auf.

Mehrere retrospektive Studien setzten sich mit der Thematik der Herztoxizität bei der thorakalen RT auseinander. Auch mittlere und niedrige Bestrahlungsdosen können eine Schädigung aller kardialer Strukturen verursachen mit variablen Zeitspannen bis zum Symptombeginn nach TRT [3]. Zum Beispiel zeigte eine gepoolte Analyse aus sechs prospektiven, dosiseskalierten NSCLC-Studien eine kumulative Erhöhung der kardialen Ereignisse [4]. Diese waren sowohl mit der Herzdosis als auch mit dem kardialen Ausgangsrisiko assoziiert. Das Risiko eines Herzereignisses bei Patienten mit einer mittleren Herzdosis (D_mean_) von <10 Gy, 10–20 Gy oder >20 Gy betrug 4 %, 7 % bzw. 21 %. Eine weitere große retrospektive Studie mit 748 NSCLC-Patienten, die eine mediane PTV-Dosis von 66,0 Gy und eine mediane Herzdosis von 12,3 Gy (D_mean_) erhielten, zeigte, dass 10,3 % der Patienten mindestens ein unerwünschtes kardiales Ereignis entwickelten nach einem medianen Intervall bis zum ersten Ereignis von 18,5 Monaten [5]. Eine weitere Studie mit 140 NSCLC-Patienten, die mit modernen RT-Techniken 60 bis 66 Gy erhielten, ergab, dass 29 % der Patienten symptomatische kardiale Ereignisse schon 15 Monate nach TRT entwickelten [6].

Zwei wichtige Fragen bleiben aber offen:

1. Da retrospektive Analysen wegen mangelnder Verfügbarkeit von Segmentierungen der kardialen Substrukturen in ihrer Aussage begrenzt sind, muss die Frage beantwortet werden, welche Substrukturen bei der Bestrahlungsplanung am relevantesten sind, wenn das Herz im Ganzen nicht geschont werden kann, und welche Dosisgrenzwerte an diesen Substrukturen des Herzens beachtet werden müssen.
2. Wie sollte man mit Patienten, die mit einem bekanntermaßen erhöhten kardialen Risiko nach TRT belastet sind, umgehen?

Letztere Frage kann momentan noch nicht abschließend beantwortet werden. Eine kardiale Überwachung asymptomatischer Patienten ist für Teilnehmer an laufenden TRT-Studien (z. B. RTOG 1308) offenbar noch nicht erforderlich. Zwischen 2016 und 2017 veröffentlichten die American Society of Clinical Oncology (ASCO), die Canadian Cardiovascular Society und die European Society of Cardiology (ESC) separate Empfehlungen für die Überwachung und Behandlung kardialer Toxizitäten von onkologischen Behandlungen einschließlich systemischer Chemotherapien und Strahlentherapie [7–9].

Eine in 2020 publizierte Umfrage setzte sich mit dem Bekanntheitsgrad der „guidelines“ (spezifisch mit der „ASCO guideline“) in der strahlentherapeutischen Community der USA auseinander [10]. Die ASCO-Leitlinie war nur 19 % der Befragten bekannt. Nach Durchsicht der Leitlinie waren nur 24 % der Radioonkologen mit den Empfehlungen einverstanden. 41 % waren der Meinung, dass die Leitlinie spezifischer sein müsste, z. B. Anhebung der maximal tolerablen Herzdosis auf 45 Gy statt 30 Gy oder Verwendung einer volumetrischen Dosisangabe und Einbeziehung früherer kardialer Risikofaktoren. Die Ergebnisse dieser Umfrage sind nicht verwunderlich, wenn man berücksichtigt, dass auf allen drei Publikationen (ASCO, CCS und ESC) insgesamt 62 Autoren aufgeführt sind, unter ihnen aber nur ein einziger Radioonkologe (als Koautor der ASCO-Leitlinie). 86 % der Befragten waren übrigens der Meinung, dass Radioonkologen bei der Herzüberwachung von Patienten mit erhöhtem RIHD-Risiko eine aktivere Rolle übernehmen sollten.

## Fazit

Anstatt auf eine irreversible und symptomatische RIHD zu warten, könnte die Nutzung des kardialen Monitorings zur proaktiven Suche nach den frühesten Anzeichen von RIHD eine Gelegenheit bieten, medizinische Interventionen zu testen, um kardiale Schäden rückgängig zu machen, abzuschwächen bzw. langfristige, irreversible RIHD zu verhindern.

In der Planung für die TRT sollte eine Herzregion definiert werden, die den rechten Vorhof, die rechte Koronararterie und die Aorta ascendens umfasst, und ein „constraint“ mit einer EQD2 von D_max_ 23 Gy vorgegeben werden.

Eine Zusammenarbeit mehrerer Disziplinen (Kardiologie, medizinische Onkologie, Radioonkologie) ist wünschenswert, um das Gesamtüberleben der Patienten weiter zu optimieren.

*Marciana-Nona Duma, Jena*
